# Substantial prevalence of enteroparasites *Cryptosporidium* spp., *Giardia duodenalis* and *Blastocystis* sp. in asymptomatic schoolchildren in Madrid, Spain, November 2017 to June 2018

**DOI:** 10.2807/1560-7917.ES.2019.24.43.1900241

**Published:** 2019-10-24

**Authors:** Lucia Reh, Aly Salimo Muadica, Pamela Carolina Köster, Sooria Balasegaram, Neville Q Verlander, Esther Ruiz Chércoles, David Carmena

**Affiliations:** 1 Parasitology Reference and Research Laboratory, National Centre for Microbiology, Majadahonda, Madrid, Spain; 2 European Program for Public Health Microbiology Training, European Centre for Disease Prevention and Control, Stockholm, Sweden; 3 These authors contributed equally; 4 Field Epidemiology Services, National Infection Service, Public Health England, London, United Kingdom; 5 Statistics, Modelling and Economics Department, National Infection Service, Public Health England, London, United Kingdom; 6 Centro de Salud María Jesús Hereza, Leganés, Madrid, Spain

**Keywords:** *Giardia*, *Cryptosporidium*, *Blastocystis*, asymptomatic children, risk analysis, Spain

## Abstract

**Background:**

Protozoan enteroparasites *Cryptosporidium* species and *Giardia duodenalis* are major contributors to the burden of gastrointestinal illness in children globally, whereas the stramenopile *Blastocystis* species has been associated with irritable bowel syndrome and skin disorders.

**Aim:**

To investigate the carriage of these parasites in voluntary asymptomatic schoolchildren (4‒14 years) in 12 different primary and secondary schools in Leganés (Madrid, Spain).

**Methods:**

In a prospective cross-sectional study, stool samples and epidemiological questionnaires on demographics and potential risk factors were collected from participating schoolchildren. Detection of enteric parasites was conducted by PCR-based methods and confirmed by sequence analysis. We calculated prevalence and odds ratios (OR) with logistic regression.

**Results:**

Stool samples and questionnaires were provided by 1,359 schoolchildren from 12 schools. The individual prevalence for any parasite was 28%; *Blastocystis* sp.*:* 13%; *G. duodenalis*: 18%; *Cryptosporidium* spp.: 1%. Two schoolchildren were infected with all three species and 53 with two species. Multivariable risk factor analysis using logistic regression models indicated that an existing infection with one parasite increased the odds for an additional infection with another parasite. The odds of *Blastocystis* sp. carriage increased up to the age of 10 years and being female increased the odds of *Cryptosporidium* spp. infection. Washing vegetables before preparing a meal was protective for *Blastocystis* sp. infection.

**Conclusion:**

We detected a larger than expected proportion of asymptomatic cases in the participanting schoolchildren. Further investigation of asymptomatic children should be considered. Good hygiene measures should be encouraged for individuals of all ages to protect from protozoal infections.

## Introduction

Enteric parasites *Cryptosporidium* spp. (Apicomplexa: Cryptosporidiidae), *Giardia duodenalis* (Metamonada: Hexamitidae) and, to a lesser extent, *Blastocystis* sp. (Stramenopiles: Blastocystidae) are important contributors to the global burden of childhood gastrointestinal illness. Clinical manifestations vary from asymptomatic carriage to acute diarrhoea and chronic disease. Children in resource-poor settings are particularly at risk with *Cryptosporidium* spp. being the second leading cause of diarrhoeal-related mortality after rotavirus in children younger than 5 years in low-income countries [1]. Cryptosporidiosis and giardiasis have been associated with impaired growth and cognitive development in low- and medium-income countries as well as in Europe [2,3]. Although the pathogenic role of *Blastocystis* sp. remains controversial [4], emerging clinical, epidemiological and laboratory evidence suggest a relationship between gastrointestinal (diarrhoea, irritable bowel syndrome) and extra-intestinal (urticarial) disorders and *Blastocystis* sp. carriage [5].

Enteric parasites also represent a serious public health concern in high-income countries, causing a considerable socioeconomic burden linked to higher income, medical and treatment costs [6]. Cryptosporidiosis and giardiasis (but not blastocystosis) are notifiable diseases in European Union and European Economic Area (EU/EEA) countries. In 2015, 10,915 and 18,031 confirmed cryptosporidiosis and giardiasis cases, respectively, were reported in the EU/EEA; infections disproportionally affected children in the age group 0‒4 years [7,8]. However, official figures may only represent a fraction of the true incidence of these diseases as symptomatic cases are often underdiagnosed and under-reported [9]. In addition, the proportion of asymptomatic carriers and subclinical infections is unknown due to limited sensitivity of conventional (e.g. microscopy) diagnostic tests and lack of large community surveys [9]. In EU/EEA countries, molecular-based assays with high diagnostic sensitivity and specificity should be the preferred method for the detection of enteric parasites in asymptomatic populations due to typically moderate to low infection rates and parasite burden [10].

In Spain, the prevalence of *Cryptosporidium* spp., *G. duodenalis* and *Blastocystis* sp. in asymptomatic, paediatric (aged < 18 years) populations have been estimated in the range of 1–35% in different community settings and regions [11-14]. However, these studies were often limited due to small sample sizes and low sensitivity of the diagnostic (mainly microscopy) methods used. Additionally, very few studies evaluated sociodemographic, environmental and/or behavioural variables that could have been associated with a higher risk of infection by enteric parasites there is a need, therefore, to better understand the epidemiology behind these parasites in Spain.

The aim of this cross-sectional study was: (i) to determine the prevalence of asymptomatic/subclinical infections by *Cryptosporidium* spp., *G. duodenalis* and *Blastocystis* sp. in a large population of schoolchildren in central Spain using PCR-based methods, and (ii) to assess potential risk and/or protective factors associated with parasite infection.

## Methods

### Study design and setting

Our study was a prospective cross-sectional study, which included molecular data on schoolchildren (4–14 years) without acute or chronic diarrhoea in the Leganés municipality (southern metropolitan area of Madrid, central Spain) between November 2017 and June 2018. Stool samples were collected from participating schoolchildren from 12 primary and secondary schools (nine public and three private) each with 180‒990 (mean: 486) schoolchildren.

In January 2017, the Leganés municipality had a total population of 21,399 schoolchildren aged 4‒14 years attending 47 public and seven private schools [15]; 51% of the schoolchildren were male. Leganés municipality was selected to allow for subsequent comparative molecular population studies between the asymptomatic schoolchildren investigated in this study and individuals of all ages with gastrointestinal symptoms attending the Severo Ochoa University Hospital located in the same area (whose faecal samples are regularly sent to the Spanish National Centre for Microbiology (Majadahonda, Spain) for parasitological screening and typing as part of an ongoing research project).

Based on previous studies in other Spanish regions, we estimated a prevalence of 3‒5% and a response rate of 30‒35% [11-13].

### Collection of samples

Schools differed by size (range: 180–990 schoolchildren) and range: (i) in eight schools the age range was 3–12 years, (ii) in three schools 3–16 years, and (iii) in one school 6–12 years; for this survey the participating age was 4-14 years. Informative meetings were held for interested families and participating schoolchildren were provided with sampling kits (sterile polystyrene plastic flask with spatula and a unique identification number) to obtain individual stool samples. Parents/legal guardians assisted in the collection of stool samples from consenting schoolchildren and brought the samples to school. Samples were collected by members of the research team at scheduled times (usually 2‒3 days after kits were provided) and transported to the Spanish National Centre for Microbiology and stored at 4 °C (1‒5 days) or − 20 °C (> 5 days) without preservatives until further diagnostic and molecular analyses.

### DNA extraction

Genomic DNA was extracted from ca 200 mg of concentrated faecal material using the QIAamp DNA Stool Mini Kit (QIAGEN, Hilden, Germany) according to the manufacturer’s instructions, except that samples mixed with InhibitEX buffer were incubated for 10 min at 95 °C. Extracted and purified DNA samples (200 µl) were kept at − 20 °C until further molecular analysis. A water extraction control was included in each sample batch processed.

### Molecular detection of *Giardia duodenalis*

*Giardia duodenalis* was detected by a real-time PCR (qPCR) method targeting a specific 62-bp region of the small subunit rRNA (*ssu* rRNA) gene of the parasite [16]. Amplification reactions were conducted in total volumes of 25 µl with 3 µl template DNA, 12.5 pmol of primers Gd-80F (5´–GACGGCTCAGGACAACGGTT–3´) and Gd-127R (5´–TTGCCAGCGGTGTCCG–3´), 10 pmol of probe (FAM–CCCGCGGCGGTCCCTGCTAG–BHQ1) and 1X TaqManGeneExpression Master Mix (Applied Biosystems, California, United States (US)). Reactions were run on a Corbett Rotor-Gene 6000 qPCR cycler (Qiagen Corbett, Hilden, Germany) using the following protocol: an initial hold step of 2 min at 55 °C, 15 min at 95 °C and 45 cycles of 15 s at 95 °C and 1 min at 60 °C. The ramping of the machine was 10 °C/s in every step. No-template water (negative) and DNA (positive) controls of genomic DNA were included in each PCR run.

### Molecular detection of *Cryptosporidium* species

*Cryptosporidium* spp. was detected using a nested-PCR protocol amplifying a 587-bp fragment of the *ssu* rRNA gene [17]. Both PCR reactions were conducted in a total volume of 50 µl including 3 μl of DNA sample and 0.3 μM of the primer pairs CRP1 (5′–CAGGGAGGTAGTGACAAGAA–3′) and CR-P2 (5′–TCAGCCTTGCGACCATACTC–3′) for the primary reaction and CR-P3 (5′–ATTGGAGGGCAAGTCTGGTG–3′) and CPB-DIAGR (5´–TAAGGTGCTGAAGGAGTAAGG–3´) for the secondary reaction. After denaturation at 94 °C for 5 min, primary and secondary PCR reactions were subjected to 35 cycles of amplification (denaturation at 94 °C for 40 s, annealing at 50 °C for 40 s, and elongation at 72 °C for 1 min), followed by a final extension at 72 °C for 10 min.

### Molecular detection of *Blastocystis* species

Detection of *Blastocystis* sp. was conducted by a direct PCR targeting *ssu* rRNA gene of the parasite [18]. The PCR reaction contained a total volume of 25 μl including 5 μl of template DNA and 0.5 μM of the primer set RD5 (5′–ATCTGGTTGATCCTGCCAGT–3′) and BhRDr (5′–GAGCTTTTTAACTGCAACAACG–3′). Amplification conditions consisted of one step of 95 °C for 3 min, followed by 30 cycles of 1 min each at 94, 59 and 72 °C, with an additional 2 min final extension at 72 °C. All the direct and nested PCR protocols described above were run on a 2720 thermocycler (Applied Biosystems). Reaction mixes included 2.5 units of MyTAQ DNA polymerase (Bioline GmbH, Luckenwalde, Germany), and 5x MyTAQ Reaction Buffer containing 5 mM dNTPs and 15 mM MgCl_2_. Laboratory-confirmed positive and negative DNA isolates for each parasitic species were used as positive controls in each PCR reaction. PCR amplicons were visualised on 2% D5 agarose gels (Conda, Madrid, Spain) and stained with Pronasafe nucleic acid staining solution (Conda). Positive-PCR products were directly sequenced in both directions using the internal primer sets by capillary electrophoresis using the BigDye Terminator chemistry (Applied Biosystems) on an on ABI 3730xl automated DNA sequencer to confirm the presence of *Cryptosporidium* and *Blastocystis* parasites. Diagnostic sensitivities of the PCR-based methods used for the detection of *G. duodenalis*, *Cryptosporidium* spp. and *Blastocystis* sp. were over 95%. Diagnostic specificities were near 100% as most of the PCR-positive samples were confirmed by sequencing.

### Questionnaire

A standardised questionnaire (Supplementary Table S1) was provided as part of the sampling kit to be completed by the children’s parents/legal guardians. Questions included: (i) demographic characteristics e.g. age, sex, country of birth and number of siblings, (ii) behavioural habits e.g. hand and fruit/vegetable washing and whether there have been any occurrence of diarrhoea in the participant, their family members, their schoolmates and/or pets, and (iii) additional questions on potential risk factors e.g. types of drinking water, whether they had swum in pools or natural waters in the 2 weeks prior to sample collection, had any contact with pets and any recent travel abroad.

Completed questionnaires, signed informed consents and individual stool samples were returned for collection by each participating schoolchild as described above, thus questionnaires were completed before parents knew the results for their child; reducing any likelihood of recall bias.

### Statistical analyses

Data entry from the epidemiological questionnaires was completed using EpiData version 4.2.0 (EpiData Association, Odense, Denmark) and analysed using Stata version 13 and 15 (STATA Corp., College Station, Texas, US). Prevalence of any *G. duodenalis*, *Cryptosporidium* spp. and *Blastocystis* sp. infections (single or multiple) in the study population were calculated. The chi-squared and/or Fisher's exact test were used to compare *G. duodenalis*, *Cryptosporidium* spp. and *Blastocystis* sp. infection rates by sex and categorical risk factors (including the other protists as a possible risk factor). A p value < 0.05 was considered evidence of statistical significance. For continuous variables, the best fitting model was explored using the likelihood ratio test for linear, quadratic, cubic and logarithmic functions. Odds ratios (OR) and their 95% confidence intervals (CI) were calculated by univariate and multivariable analyses using logistic regression models to assess the association between potential risk factors and infections with each of these parasites. Exact logistic regression was used to calculate univariate odds ratios to avoid division by zero. Factors with a p value of 0.2 or less from the univariate analysis were selected for possible inclusion in the multivariable logistic regression model. Variables with the lowest p value were added to the model in a forward stepwise approach and used to test for possible confounders (indicated by a change of > 20% in the OR of other factors) and interactions. To account for possible non-independence at school and family levels, multilevel models with school and family nested within a school as random effects were compared to the logistic model with other variables as fixed effects. If variable inclusion was not significant using the likelihood ratio test, it was not included in the model and other variables were re-checked. Thus, for the multivariable analysis, only adjusted odds ratios (aOR) are displayed for variables that were included in the final model.

### Ethical statement

Written informed consent was obtained from parents/legal guardians of participating schoolchildren. Socio-demographic, epidemiological data and stool samples were coded by a unique identifier and were only published in an aggregated form to protect the identity of the schoolchildren. All study procedures, informed consent forms and epidemiological questionnaires were approved by the Ethics Committee of the Health Institute Carlos III under the reference number CEI PI 17_2017-v3.

## Results

### Prevalence of parasites

In this study, 1,356 stool samples and corresponding questionnaires were collected from asymptomatic schoolchildren attending one of 12 schools in Leganés (Madrid, Spain). Of these, 738 (54%) were male and 618 (46%) were female. The age of the participating schoolchildren ranged from 4 to 14 years with a median age of 7 years.

**Figure 1:**
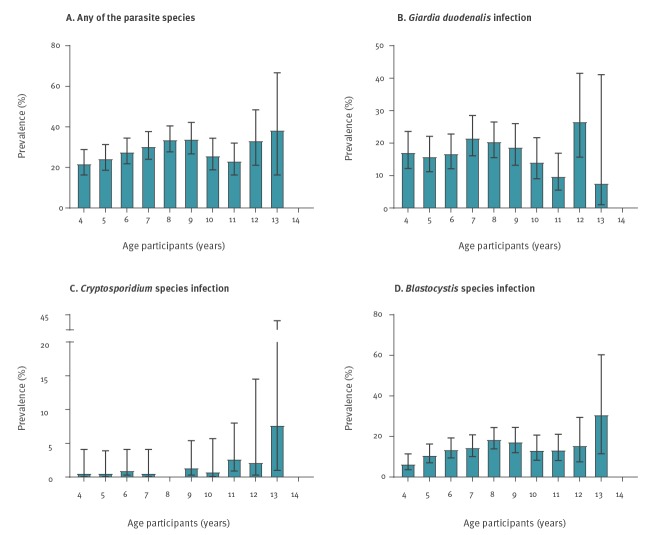
Frequency of infection in surveyed asymptomatic schoolchildren, by age, Madrid, November 2017–June 2018 (n = 1,359)

The median age that schoolchildren were infected with *Blastocystis* sp. was 8 years, for *Cryptosporidium* spp. 9 years and for *G. duodenalis* 7 years (Figure 1). Of 1,356 participating schoolchildren, 113 (8%) were born outside Spain of which, 106 (94%) were born outside the EU. These rates did not differ between infected and uninfected schoolchildren; both groups had a median of one sibling.

The number of schoolchildren differed between the 12 participating. The mean participation rate was 22% (range: 12─ 47%).

**Figure 2:**
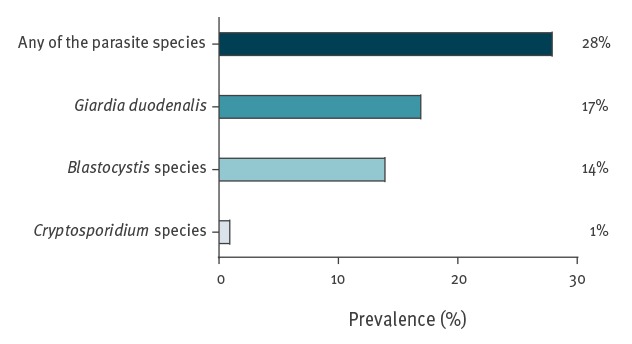
Overall prevalence of *Giardia duodenalis*, *Cryptosporidium* spp. and *Blastocystis* sp. infections in surveyed asymptomatic schoolchildren, Madrid, November 2017–June 2018 (n = 1,359)

Of 1,359 samples, one parasite species was detected in 382 (28%); 237 children (17%) were infected with *G. duodenalis*, 187 (14%) with *Blastocystis* sp. and 13 (1%) with *Cryptosporidium* spp. (Figure 2).

Multiple infections with two parasite species were found in 53 samples (4%) of which, 47 (3%) were co-infected with *Blastocystis* sp. and *G. duodenalis*, five (0.4%) with *Blastocystis* sp. and *Cryptosporidium* spp. and one (0.1%) with *G. duodenalis* and *Cryptosporidium* spp. One sample (0.1%) was infected with all three parasite species.

**Figure 3:**
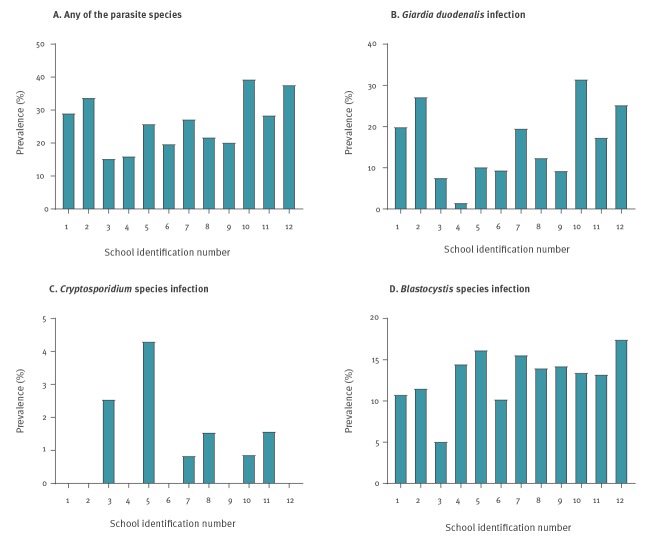
Prevalence of *Giardia duodenalis*, *Cryptosporidium* spp. and *Blastocystis* sp. in surveyed asymptomatic schoolchildren, by school, Madrid, November 2017–June 2018 (n = 1,359)

Prevalence rates between schools ranged between 15–40% for any enteroparasite, 2–32% for *G. duodenalis,* 5–18% for *Blastocystis* sp. and 0–4% for *Cryptosporidium* spp. (Figure 3).

### Risk factor analysis: univariate analysis

Results of the univariate analysis are summarised in Table 1, with adjustment for school (*G.* *duodenalis* and *Cryptosporidium* spp.) and family (*G.* *duodenalis* only) as this was seen to be important in the final model for these infections. Infection with one enteroparasite species increased the odds for an additional infection after adjusting for school and family where necessary (Table 1).

**Table 1:** Distribution of protozoan infections among schoolchildren, by risk factor, Madrid, November 2017–June 2018 (n = 1,356)

Exposure	*Blastocystis* sp. (n = 187)				*Giardia duodenalis* ^a^ (n = 237)				C*ryptosporidium* spp.^a^ (n = 13)			
	N	%	OR (95% CI)	p value	n	%	OR (95% CI)	p value	n	%	OR (95% CI)	p value
*Blastocystis* sp. infection	NA	NA	NA	NA	48	20.3	2.20 (1.29–3.78)	0.004	6	46.2	5.46 (1.77–16.81)	0.003
*Giardia* *duodenalis* infection	48	25.7	1.79 (1.22‒2.60)	0.001	NA	NA	NA	NA	2	15.4	1.11 (0.24–5.25)	0.893
*Cryptosporidium* spp. Infection	6	3.2	5.50 (1.51‒19.32)	0.001	2	0.8	1.59 (0.21–12.03)	0.653	NA	NA	NA	NA
Female	84	44.9	0.97 (0.70‒1.34)	0.846	107	45.2	1.14 (0.77–1.69)	0.50	10	76.9	3.89 (1.05–14.36)	0.023
Age (by increasing year)	NA	NA	1.09 (1.02‒1.16) Square age 0.97(0.94-0.99)	0.011 0.018	NA	NA	0.95 (0.87–1.04)	0.183	NA	NA	1.27 (1.01–1.60)	0.045
Foreign origin	21	11.2	1.53 (0.88‒2.56)	0.095	16	6.8	1.00 (0.46–2.19)	0.99	2	15.4	2.11 (0.42–10.52)	0.362
Non-European origin	19	10.2	1.45 (0.81‒2.49)	0.160	16	6.8	1.19 (0.46–3.07)	0.72	2	15.4	4.01 (0.74–21.78)	0.107
Having siblings	148	79.1	1.21 (0.79‒1.91)	0.365	187	78.9	1.03 (0.75–1.39)	0.871	13	100	1.33 (0.86–2.02)	0.182
Contact with infants	26	13.9	0.85 (0.52‒1.34)	0.475	32	13.5	0.78 (0.45–1.36)	0.381	1	7.7	0.33 (0.42–2.60)	0.293
Recent travel	33	17.7	1.08 (0.69‒1.63)	0.727	43	18.1	1.05 (0.61–1.80)	0.871	2	15.4	1.02 (0.22–4.72)	0.984
Recent travel to EU countries	12	6.4	1.22 (0.59‒2.35)	0.535	10	4.2	0.50 (0.18–1.45)	0.202	2	15.4	3.19 (0.34–15.00)	0.116^c^
Diarrhoea in past week	5	2.7	0.55 (0.17‒1.40)	0.207	6	2.5	0.45 (0.15–1.38)	0.163	0	0.0	0.00 (0.00–6.37)	0.405^c^
Diarrhoea in family	29	15.5	1.15 (0.72‒1.79)	0.518	27	11.4	0.78 (0.42–1.45)	0.212	3	23.1	1.48 (0.39–5.60)	0.56
Diarrhoea in schoolmates	14	7.5	1.31 (0.42‒4.87)	0.620	14	5.9	0.79 (0.27–2.30)	0.665	2	15.4	0.94 (0.08–10.96)	0.946
Diarrhoea in pets	4	2.1	0.47 (0.12‒1.29)	0.138	11	4.6	1.21 (0.47–3.11)	0.699	2	15.4	4.76 (0.92–24.65)	0.063
Contact with cats	13	7.0	0.72 (0.36‒1.32)	0.279	19	8.0	0.78 (0.38–1.64)	0.543	0	0.0	0.00 (0.00–2.93)	0.251^c^
Contact with dogs	28	15.0	0.99 (0.62‒1.55)	0.978	28	11.8	0.57 (0.32–1.05)	0.071	3	23.1	1.65 (0.44–6.18)	0.454
Handwashing	167	89.3	1.31 (0.79‒2.27)	0.278	207	87.3	1.17 (0.65–2.13)	0.60	9	69.2	0.40 (0.12–1.35)	0.139
Vegetable washing	180	96.3	0.29 (0.11‒0.88)	0.006	232	97.9	1.78 (0.32–9.91)	0.513	13	100	0.27 (0.04-∞)^b,c^	1.000
Consumption of bottled water	37	19.8	0.84 (0.56‒1.25)	0.379	49	20.7	0.94 (0.58–1.53)	0.803	2	15.4	0.69 (0.15–3.12)	0.637
Consumption of spring water	15	8.0	1.70 (0.87‒3.13)	0.075	16	6.8	1.11 (0.47–2.61)	0.809	0	0.0	0.00 (0.00–5.27)	0.391^c^
Swimming in pools	73	39.0	1.07 (0.77‒1.49)	0.673	93	39.2	0.97 (0.62–1.51)	0.577	2	15.4	0.30 (0.07–1.44)	0.135
Swimming in natural water	1	0.5	1.56 (0.03‒15.87)	0.689	1	0.4	1.90 (0.01–37.48)	0.674	0	0.0	0.00 (0.00–84.31)	0.825^c^

CI: confidence interval; EU: European Union; NA: not available; OR: odds ratio.

^a^ Results for *Giardia duodenalis* were adjusted for school and family. Results for *Cryptosporidium* spp. were adjusted for school.

^b^ OR and 95% CI were calculated with exact logistics. p value was derived from two-sided Fisher’s exact test.

^c^ Without adjustment (no model convergence of no result for CI/p values).

Participants carrying *Blastocystis* sp. had significantly higher odds of being infected with *G. duodenalis* (OR: 1.79; 95% CI: 1.22‒2.60) and *Cryptosporidium* spp. (OR: 5.50; 95% CI: 1.51‒19.32) and vice versa. There was no significant association between *G. duodenalis* and *Cryptosporidium* spp. infections. Age was a risk factor for *Blastocystis* sp. Carriage with a quadratic relationship (OR: 1.09 linear increase per year; 95% CI: 1.02‒1.16, with an OR of 0.97; 95% CI: 0.94–0.97 for the squared term, peaking at age 10) and *Cryptosporidium* spp. infection (OR increase per year: 1.27; 95% CI: 1.00‒1.60). Being female increased the odds of *Cryptosporidium* spp. infection (OR: 3.89; 95% CI: 1.05‒14.35). Washing of vegetables before meal preparation was a protective factor against *Blastocystis* sp. carriage (OR: 0.29; 95%CI: 0.11‒0.88).

### Risk factor analysis: multivariable analysis (MVA)

In the adjusted multivariable model for *Blastocystis* sp. carriage, factors that significantly increased the odds of carriage were infection with *Cryptosporidium* spp. (aOR: 5.84; 95% CI: 1.90‒17.98) and *G. duodenalis* (aOR: 1.81; 95% CI: 1.25‒2.62). Age was related as a quadratic function with an aOR for the linear term of the quadratic of 1.85 (95% CI: 1.18‒2.92) and an aOR for the square of age 0.97 (95% CI: 0.94‒0.99) with a peak at age 10 years. Vegetable washing was found to be a protective factor against *Blastocystis* carriage in the MVA analysis (aOR: 0.30; 95% CI: 0.12‒0.78). (Table 2).

**Table 2:** Multivariate analysis of distribution of protozoan infections among schoolchildren, by risk factor, Madrid, November 2017–June 2018

Exposure	*Blastocystis* sp.	*Giardia duodenalis*	*Cryptosporidium* spp.
	aOR (95% CI)	aOR (95% CI)	aOR (95% CI)
*Blastocystis* sp. infection	NA	2.20 (1.29‒3.78)	5.80 (1.86‒18.13)
*Giardia duodenalis* infection	1.81 (1.25‒2.62)	NA	NA
*Cryptosporidium* spp. infection	5.84 (1.90‒17.98)	NA	NA
Female	NA	NA	4.16 (1.11‒15.59)
Age by (years)^a^	Age 1.85 (1.18‒2.92), squared 0.97 (0.94‒0.99)	NA	NA
Vegetable washing	0.30 (0.12‒0.78)	NA	NA

aOR: adjusted odds ratio; CI: confidence interval; NA: not available.

^a^ Quadratic function, inverted U peak age 10 years.

*G. duodenalis* and *Cryptosporidium* spp. infection did not change the effect or magnitude of other risk factors as the model was also tested without them.

The final adjusted multivariable model for *G. duodenalis* infection only contained *Blastocystis* sp. carriage as a risk factor (aOR: 2.20; 95% CI: 1.29‒3.78) but was adjusted for the variation between schools and family clustering (Table 2). For *Cryptosporidium* spp. infection, the final adjusted multivariable model contained *Blastocystis* sp. carriage (aOR: 5.80; 95% CI: 1.86–18.13) and being female (aOR: 4.16; 95% CI: 1.11‒15.59) as risk factors and was adjusted for the variation between schools.

When *Blastocystis* sp. was excluded from the model, age became an additional risk factor for *Cryptosporidium* spp. infection, possibly due to the strong association of *Blastocystis* sp. carriage and increasing age. (Table 2).

## Discussion

The prevalence of enteric parasite species of public health relevance has been studied in a variety of symptomatic [1] and asymptomatic [19,20] children often living in low-income countries. However, studies focusing on high-income countries like Spain are less common. In this cross-sectional study, we investigated the asymptomatic carriage of *G. duodenalis*, *Cryptosporidium* spp. and *Blastocystis* sp. in a large paediatric population in central Spain. Our findings suggest that subclinical infections by these parasites are more common than previously anticipated, indicating that otherwise healthy schoolchildren may play an important role in the spreading of these disease at the community level.

### Prevalence data

Our PCR-detected prevalence in the investigated asymptomatic schoolchildren population was higher than expected with 28% of the children carrying at least one parasite species; *G. duodenalis* infection was the most frequent (17%) followed by *Blastocystis* sp. carriage (14%) and *Cryptosporidium* spp. (1%). The prevalence of co-infections was 4%, with *Blastocystis* sp. and *G. duodenalis* being the most frequent combination (3%). These findings were in line with those previously reported in Spanish immunocompetent children in both rural and urban areas, where *G. duodenalis* was detected in the range of 3‒36% in the provinces of Álava [14], Ávila [21], Cuenca [22], Madrid [23], Salamanca [24,25] and Valencia [26]. Infections by *Cryposporidium* spp. were identified in 1‒10% of children in Álava and Salamanca [14,24,25], whereas *Blastocystis* sp. was found at 5‒20% infection rates in schoolchildren from Álava [14], Salamanca [27] and Valencia [26]. The reported prevalence in all these studies was based on conventional, less sensitive methods (mainly microscopy) and most of them focused on children of young age only. Therefore, the data presented here may represent an improvement in terms of diagnostic accuracy.

A range of comparatively lower infection rates has been reported in similar European molecular epidemiological studies. For example, the prevalence rate for *G. duodenalis* has been found in the range of 1‒7% in children attending day care in the Netherlands [28,29] and United Kingdom (UK) [30], in children attending national vaccination programs in Portugal [31] and in asymptomatic immigrant children in Italy [32]. For *Blastocystis* sp., a range of PCR-based prevalence has been reported from asymptomatic study populations from France and Ireland. For the former, a study identified a *Blastocystis* sp. prevalence rate of 16.2% in asymptomatic carriers in France [33], while numbers reported from a study in Ireland were as high as 56% [34]. Our prevalence rate was lower, which might be because our study only included schoolchildren aged 4‒14 years and was dependent on schools that participated in Leganés. The prevalence of asymptomatic *Cryptosporidium* spp. infections in pre-school children (age 1–40 months) in the UK was identified as 1.3% [30], lower than the 3% reported in in the Netherlands [29]. However, caution should be taken when comparing prevalence results from different studies due to the variation in factors e.g. composition of the study population, the detection methods for the different parasites (e.g. microscopy vs PCR-based methods), the geographic area considered or the sampling period.

While clinical infections with *G. duodenalis* and *Cryptosporidium* spp. have been reported, primarily for children aged 1‒3 years [9], we detected asymptomatic infections by both parasites in schoolchildren of all age groups investigated. We found that the median age of the schoolchildren was 7 years, the median age of schoolchildren infected/colonised with *G. duodenalis* and *Blastocystis* sp. was 8 years, while the median age for *Cryptosporidium* spp. was 9 years. This could suggest that subclinical infection with these parasites is not restricted to a certain age group within the paediatric population. It is important to highlight that the majority of previously published reports have focused on notifiable giardiasis and cryptosporidiosis cases in infants (age 0‒12 months) and toddlers (age 13‒36 months) with clinical symptoms. It is possible that in older children with a more mature immune system, *G. duodenalis* and *Cryptosporidium* spp. infections are more frequently asymptomatic and, therefore, less frequently diagnosed. Previous research conducted after outbreak investigations [35] or in hyper-endemic scenarios [36] have shown that earlier infections with both enteric parasites can elicit a protective host immune response leading to a reduced risk of re-infection and reduced development of overt symptoms in secondary infections. Other factors that may be involved in the outcome of the infection include the genotype of the parasite [37] or the richness and diversity of the gut microbiota [38]. Better identification of the underlying causes for subclinical infection in older schoolchildren (age >36 months) is important as they might serve as asymptomatic, thus unnoticed, carriers spreading the infection to more vulnerable populations e.g. younger children, the elderly (aged 65 years and older) or immunocompromised individuals.

Our study recruited children from 12 different schools with a differing number of schoolchildren, differing geographic distribution within the municipality of Leganés, differing participation rate and different funding source (public vs private). We observed that prevalence of enteroparasite species differed among the investigated schools, most notably for *G. duodenalis* for which the prevalence rates ranged between 1.6% and 31.6%. These data suggest that local clusters of asymptomatic infections and school transmission may be occurring at some of the schools; a notion previously investigated in households with preschool children in the Netherlands [39] and the UK [40]. The underlying causes of the high local prevalence rates reported here should be the subject of future investigations.

### Risk factor analysis

Univariate and multivariable risk factor analyses revealed an association between the primary and the secondary infection with the investigated parasites. In the final adjusted multivariable model, *G. duodenalis* and *Cryptosporidium* spp. were risk factors for *Blastocystis* sp. carriage and vice versa, but no association between *G. duodenalis* and *Cryptosporidium* spp. infections were observed. This may suggest that *Blastocystis* sp. colonisation alters the susceptibility to other protozoa. Similarly, a study from France identified higher *Blastocystis* sp. prevalence in patients that were infected with other enteric protozoa [33]. Co-infections of *Blastocystis* sp. and *G. duodenalis* were also identified in 4% of asymptomatic schoolchildren in Lebanon [20] and 19.6% of indigenous children from the Colombian Amazon basin [41].

The pathogenic role of *Blastocystis* sp. is widely disputed in the field. While some studies describe *Blastocystis* sp. as a harmful parasite associated with intestinal (diarrhoea, irritable bowel syndrome) and extra-intestinal (urticaria) disorders [4], other publications consider *Blastocystis* sp. as a commensal that is part of the healthy gut flora [34]. In our analysis, increasing age up to the age of 10 years increased the likelihood of *Blastocystis* sp. carriage, after which the risk decreased. However, this association could also be explained by an incomplete or missing clearance of *Blastocystis* sp. from the gut system after initial colonisation and subsequent accumulation throughout life [14]. This would favour the commensal nature of the parasite. In contrast, a French study involving 788 clinical patients from 11 hospitals detected a significantly lower mean age in *Blastocystis* sp. positive individuals than in negative individuals [33].

We found a higher odds of *Cryptosporidium* spp. infections in females, which is surprising as there is no obvious biological or socio-behavioural explanation. Therefore, this association may be caused by an undetected underlying confounding factor. Significant differences in infection rates between males and females have been found in some European studies [42].

In our adjusted multivariable model, we have only identified that hand washing was a protective factor against *Blastocystis* sp. colonisation. This finding is not surprising as poor hygiene has previously been found to increase the risk of infection with different enteroparasites [43]. However, this study was conducted in a low-income country where access to clean water and sanitation is more restricted than in a developed country like Spain. Additionally, in compliance with the European legislation, Spain has strict food and water laws and safety regulations in place. Also, self-administered questionnaires about hygiene measures may contain a certain degree of recall and reporting bias as hygiene practices may be recorded too positively.

### Strengths and limitations

The main strength of this study is the high number of participating schoolchildren and number of samples analysed, which allowed for a robust analysis of prevalence and associated risk factors.

The results obtained in this cross-sectional molecular epidemiological study may have been potentially biased by some methodological issues: (i) schools chose to participate in the study and although we managed to obtain a spread of 12 schools (private/mostly public, wide age range), we cannot surmise that these are representative of Leganés, (ii) as the study was voluntary, it is possible that families at higher risk of infection were more likely to participate. The distorting effect of this behaviour would have been more apparent in schools with lower participation rates. Conversely, the ‘worried well’ (people who are healthy but are concerned about becoming ill) may also be more willing to participate, (iii) questionnaire data may have resulted in some recall/recording bias, and (iv) unnoticed seasonal variations of the parasites investigated could have been missed as could the synergistic effect of other viral, bacterial or parasitic species.

### Conclusions

In our study, we found that potentially harmful enteric parasites, mainly *G. duodenalis*, are common in otherwise healthy schoolchildren in central Spain. These findings highlight the relevance of asymptomatic carriers in the dissemination of diarrhoea-causing pathogens at the community level. Unnoticed school and household transmission events appear to be evident from this study and could represent a public health issue for at-risk populations i.e. young children, the elderly and immunocompromised individuals. Implementing and reinforcing preventive measures (e.g. handwashing, disinfection procedures) are the most cost-effective actions to minimise the risk of infection by these parasites.

## Supplementary Data

320000 Supplementary Table S1
