# Supplementary material for: Substantial prevalence of enteroparasites Cryptosporidium spp., Giardia duodenalis and Blastocystis sp. in asymptomatic schoolchildren in Madrid, Spain, November 2017 to June 2018
Source: Euro Surveill. 2019 Oct 24;24(43):1900241. doi: 10.2807/1560-7917.ES.2019.24.43.1900241 (PMC6820128; doi:10.2807/1560-7917.ES.2019.24.43.1900241)
Supplement: Supplementary Table S1 [file 1900241_CARMENA_SupplementaryTableS1.pdf]

Supplementary Table S1. **English version of the standardised epidemiological questionnaire used in this study.**

This supplementary material is hosted by Eurosurveillance as supporting information alongside the article [Substantial prevalence of protist enteroparasites *Cryptosporidium* spp., *Giardia duodenalis* and *Blastocystis* sp. in asymptomatic school children in Leganés, Madrid (Spain), November 2017-June 2018] on behalf of the authors who remain responsible for the accuracy and appropriateness of the content. The same standards for ethics, copyright, attributions and permissions as for the article apply. Supplements are not edited by *Eurosurveillance* and the journal is not responsible for the maintenance of any links or email addresses provided therein.

**Variable**	**Category and Stata code**
School sampled	String variable (free text)
Sampling kit ID	String variable (free text)
Sampling date	Date variable
Locality	String variable (free text)
Country of birth	String variable (free text)
Sex	Male (0)<br>Female (1)
Age	Integer variable (number)
Number of siblings	Integer variable (number)
Diarrhoea in the past 7 days?	No (0)<br>Yes (1)
Contact with children <3 years?	No (0)<br>Yes (1)
Diarrhoea in family members in the last month?	No (0)<br>Yes (1)
Diarrhoea in school mates in the last month?	No (0)<br>Yes (1)<br>I don’t know (2)
Traveling abroad in the past 6 months?	No (0)<br>Yes (1)
Traveling abroad in the past 6 months to non-EU countries?	No (0)<br>Yes (1)
Which countries	String variable (free text)
Regular contact with dogs at home?	No (0)<br>Yes (1)
Regular contact with cats at home?	No (0)<br>Yes (1)
Cats/ dogs with diarrhoea at home in the past 6 months?	No (0)<br>Yes (1)<br>I don’t know (2)
Source of drinking water?	Tap water (0)<br>Bottled water (1)<br>Tap water and bottled water (2)<br>Tap water, bottled water and fountains/springs (3)<br>Tap water and fountains/springs (4)<br>Other (5)
Practiced water sports?	No (0)<br>Pools (1)<br>Natural waters (2)<br>Both (3)
Practiced hand washing before meals?	Always (0)<br>Habitually (1)

	Rarely (2)
Practiced washing of vegetables, fruits?	Always (0)<br>Habitually (1)<br>Rarely (2)
